# Impact of Atrial Fibrillation with Rapid Ventricular Response on Atrial Fibrillation Recurrence: From the CODE-AF Registry

**DOI:** 10.3390/jcm13185469

**Published:** 2024-09-14

**Authors:** Joo Hee Jeong, Yong-Soo Baek, Junbeom Park, Hyung Wook Park, Eue-Keun Choi, Jin-Kyu Park, Ki-Woon Kang, Jun Kim, Young Soo Lee, Jin-Bae Kim, Jong-Il Choi, Boyoung Joung, Jaemin Shim

**Affiliations:** 1 Division of Cardiology, Department of Internal Medicine, Korea University Anam Hospital, Korea University College of Medicine, Seoul 02841, Republic of Korea; jessica0115@naver.com (J.H.J.); jongilchoi@korea.ac.kr (J.-I.C.); 2 Department of Internal Medicine, Inha University Hospital, Inha University College of Medicine, Incheon 22332, Republic of Korea; existsoo@hanmail.net; 3 Department of Cardiology, College of Medicine, Ewha Womans University, Seoul 07804, Republic of Korea; parkjb@ewha.ac.kr; 4 Department of Cardiology, Chonnam National University Hospital, Chonnam National University School of Medicine, Gwangju 61469, Republic of Korea; mdhwp@chonnam.ac.kr; 5 Department of Internal Medicine, Seoul National University Hospital, Seoul National University College of Medicine, Seoul 03080, Republic of Korea; choiek417@gmail.com; 6 Department of Cardiology, Hanyang University Seoul Hospital, Hanyang University College of Medicine, Seoul 04763, Republic of Korea; cardiohy@gmail.com; 7 Division of Cardiology, Chung-Ang University Hospital, Chung-Ang University College of Medicine, Seoul 06973, Republic of Korea; kwkang0115@gmail.com; 8 Heart Institute, University of Ulsan College of Medicine, Asan Medical Center, Seoul 05505, Republic of Korea; mdjunkim@gmail.com; 9 Division of Cardiology, Department of Internal Medicine, Daegu Catholic University Medical Center, Catholic University of Daegu, Daegu 42472, Republic of Korea; mdleeys@cu.ac.kr; 10 Division of Cardiology, Department of Internal Medicine, Kyung Hee University Hospital, Kyung Hee University, Seoul 02453, Republic of Korea; jinbbai@khu.ac.kr; 11 Division of Cardiology, Department of Internal Medicine, Severance Cardiovascular Hospital, Yonsei University College of Medicine, Seoul 03722, Republic of Korea

**Keywords:** atrial fibrillation, ventricular rate, rapid ventricular response, rhythm control

## Abstract

**Background/Objectives**: Relatively little has been established about the association of rapid ventricular response (RVR) with further recurrence of atrial fibrillation (AF). This study investigated the impact of RVR on the recurrence of AF. **Methods**: Data were obtained from a multicenter, prospective registry of non-valvular AF patients. RVR was defined as AF with a ventricular rate > 110 bpm. The primary endpoint was the recurrence of AF, defined as the first AF detected on 12-lead electrocardiography during follow-up. Secondary endpoints included manifestation of AF during follow-up and major adverse cardiovascular events (MACEs), a composite of thromboembolic events, major bleeding, myocardial infarction, and death. **Results**: Among 5533 patients, 493 (8.9%) presented RVR. Patients with RVR were younger, had smaller left atrial diameters, and more frequently had paroxysmal AF. During the mean follow-up duration of 28.6 months, the RVR group exhibited significantly lower recurrence of AF (hazard ratio: 0.58, 95% confidence interval: 0.53–0.65, *p* < 0.001). There was no significant difference in the occurrence of MACEs between patients with RVR and those without RVR (0.96, 0.70–1.31, *p* = 0.800). AF with RVR was identified as an independent negative predictor of AF recurrence (0.61, 0.53–0.71, *p* < 0.001). **Conclusions**: In patients with AF, those with RVR had a significantly lower recurrence of AF without an increase in MACEs. RVR is a favorable marker that may benefit from early rhythm control.

## 1. Introduction

Atrial fibrillation (AF) imposes a significant health burden on patients and the health care system [1,2]. Rhythm control is defined as an attempt to restore and maintain sinus rhythm, which is indicated to reduce AF burden and improve AF-related symptoms and quality of life. Recent advancements in ablation strategies have highlighted the importance of early rhythm control in patients with AF [3,4]. However, the beneficial effects of rhythm control might differ according to patient demographics, comorbidities, and AF characteristics, which implies an appropriate selection of patients that might benefit most from rhythm control [1,2]. AF with rapid ventricular response (RVR) is a commonly used term for AF with a fast ventricular rate (>110 bpm). During AF with RVR, irregular and fast ventricular rates reduce ventricular filling and stroke volume, contributing to patient-related symptoms and the development of heart failure [5,6]. Accordingly, rate control is an integral part of AF management and can effectively improve AF-related symptoms and heart failure hospitalization. Recent guidelines recommend lenient control of heart rate (<110 bpm) for AF with a rapid ventricular rate [2,7]. 

Although rate control is an initial approach for AF with a rapid ventricular rate, less has been established about the impact of AF with RVR on clinical outcomes related to rhythm control. In patients with AF and RVR, conversion to sinus rhythm is often observed in a short time. Spontaneous sinus conversion is associated with an increased atrial fibrillatory rate, while its association with ventricular rate has been less well explored [8]. Based on a multicenter prospective registry of patients with non-valvular AF (COmparison study of Drugs for symptom control and complication prEvention of Atrial Fibrillation [CODE-AF]), this study aimed to investigate the impact of AF with RVR on the recurrence of AF and maintenance of sinus rhythm.

## 2. Materials and Methods

### 2.1. Study Design and Population

The CODE-AF registry is an ongoing, prospective, multicenter, observational study of adult patients with non-valvular AF in South Korea. Patients were enrolled from 19 tertiary centers across all geographical regions of Korea, including 12,664 patients from June 2016 to February 2023. Patients were included if they were diagnosed as non-valvular AF patients and aged older than 18 years. AF was defined as clinical AF recorded by 12-lead electrocardiography (ECG) or an ECG strip of AF ≥ 30 s that was confirmed by an electrophysiologist. Patients not eligible for maintaining anticoagulation for non-valvular AF were excluded: (i) pregnant or breast-feeding women, (ii) patients whose expected survival was less than 1 year, (iii) patients with transient AF caused by reversible conditions (including post-operative AF within 3 months after cardiac surgery, hyperthyroidism, and pulmonary embolism), and (iv) patients who required chronic anticoagulation for valvular AF. Written informed consent was obtained from all patients or legal representatives. The protocols of the CODE-AF registry conformed to the principles of the Declaration of Helsinki and were approved by the institutional review board of Korea University Anam Hospital. This study was registered at ClinicalTrials.gov (NCT02786095). Detailed protocols and further information regarding data collection for the CODE-AF registry have been published previously [9].

**Figure 1:**
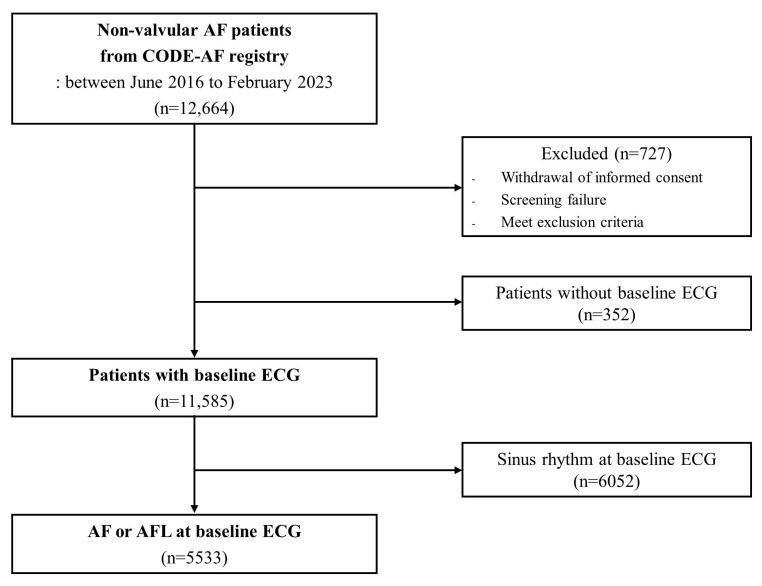
Flowsheet of the study. AF, atrial fibrillation; ECG, electrocardiography; AFL, atrial flutter.

A total of 12,664 patients with non-valvular AF were enrolled between June 2016 and February 2023 (Figure 1). Among them, 727 were excluded due to (i) withdrawal of informed consent, (ii) screening failure, or (iii) meeting the exclusion criteria. Patients without baseline ECG (n = 352) and those with sinus rhythm on baseline ECG (n = 6052) were excluded; analyses were conducted on 5533 patients with AF or atrial flutter at baseline ECG. 

### 2.2. Outcome Measurement and Definition of Variables

The primary endpoint was the recurrence of AF during follow-up, defined as the first AF detected on 12-lead ECG during follow-up. Secondary endpoints included (i) manifestation of AF at 6 months follow-up, (ii) manifestation of AF at 12 months follow-up, (iii) manifestation of AF at 36 months follow-up, (iv) AF-related hospitalization, and (v) major adverse cardiovascular events (MACEs). Patients were followed up with clinical outcomes every 6 months, either through regular clinic visits or telephone contact. If the patient visited the clinic, a follow-up 12-lead ECG was performed. 

AF with RVR was defined as AF with a ventricular rate of >110 beats per minute. Patients were classified into two groups (RVR group and No RVR group) according to their heart rates at baseline ECG. AF-related hospitalization included hospitalization due to aggravation of symptoms or heart failure and elective admission for rate control or rhythm control. Presence or recurrence of AF was judged by the 12-lead ECG during follow-up. MACE was defined as a composite outcome of thromboembolic events (ischemic stroke, systemic thromboembolism, or transient ischemic attack), major bleeding (defined according to the International Society on Thrombosis and Hemostasis criteria [10]), myocardial infarction, and death. Atrial flutter at baseline ECG is not equivalent to isolated atrial flutter without AF—all patients had a previous diagnosis of AF before enrollment. Heart failure was defined according to the contemporary guideline—heart failure with reduced ejection was narrowed down to those with a left ventricular ejection fraction < 40% [11]. The detailed definitions of the variables are provided in Supplementary Table S1. 

### 2.3. Statistical Analysis

Categorical variables are described as numbers and percentages, and continuous variables are described as means and standard deviations. The Student’s *t*-test, the Mann–Whitney U test, the chi-square test, or Fisher’s exact test were used to compare variables, as indicated. One-way analysis of variance was used to compare multiple variables. Kaplan–Meier analysis and log-rank tests were used to assess time-dependent variables. The Cox proportional hazards model was used, and variables that were statistically significant were adjusted for multivariable Cox regression analysis. Missing patterns of independent variables were examined, and variables with missing data < 1% were considered for the multivariable analysis—data with missing values were excluded from analysis. Left atrial diameter and left ventricular ejection fraction had more missing values but were included for multivariable analysis regarding its clinical significance. For primary or secondary endpoints, data that lacked dependent variables were excluded from the analysis. For the primary endpoint, the proportional hazards assumption was assessed by Schoenfeld residuals, and no violation was observed. To reduce selection bias, a propensity score matching analysis was conducted to compare patients with and without RVR. For the propensity score matching analysis, the likelihood of RVR was quantified using a multivariable logistic regression model. All previously specified baseline characteristics were included for propensity score matching: age, sex, systolic and diastolic blood pressure, body mass index, CHA_2_DS_2_-VASc score, HAS-BLED score, comorbidities, paroxysmal AF, atrial flutter at baseline, AF-related symptom, medications, any rhythm control, left atrial diameter, and left ventricular ejection fraction. After computing the expected probabilities, we matched each patient in the RVR group with those in the No RVR group at a 1:1 ratio using the nearest neighbor method. A caliper width equal to 0.2 times the standard deviation of logit propensity matching was chosen to minimize mean square error [12]. The balance of baseline features between the RVR and No RVR groups was examined using the standardized mean difference. A standardized mean difference of <0.1 indicated a negligible difference (Supplementary Figure S1). Sensitivity analysis was performed, which excluded (i) patients who received rhythm control (Cohort A) and (ii) patients with persistent AF (Cohort B). All tests were two-tailed, and statistical significance was defined as a *p*-value ≤ 0.05. All statistical analyses and model developments were performed using the SPSS software (version 26; SPSS Inc., Chicago, IL, USA) and R Statistical software (version 4.2.3; R Foundation for Statistical Computing, Vienna, Austria).

## 3. Results

### 3.1. Baseline Characteristics

Among 12,664 patients with non-valvular AF, 5533 patients with AF or atrial flutter at baseline ECG were eligible for analysis. The baseline characteristics of the study population are shown in Table 1. The mean age at enrollment was 68.1 ± 10.6 years, and 3689 (66.7%) of the total patients were male. The mean CHA_2_DS_2_-VASc score was 2.7 ± 1.7, and 2402 patients (45.2%) had paroxysmal AF. Rate control medications were prescribed to 3709 (67.0%) patients. Rhythm control was attempted in 2313 patients (41.8%), including previous catheter ablation (n = 411, 7.4%), previous direct current cardioversion (n = 759, 13.7%), and antiarrhythmic drugs (n = 1778, 32.1%). 

Among 5533 patients, 493 (8.9%) had a ventricular rate > 110 beats per minute (RVR group). Compared with the No RVR group, the RVR group revealed younger age (66.9 ± 11.4 vs. 68.1 ± 10.4, *p* = 0.018) and lower proportions of male sex (59.2 vs. 67.4%, *p* < 0.001), implantable cardioverter-defibrillator implantation (0.2 vs. 1.0%, *p* = 0.001), history of stroke or transient ischemic attack (11.8 vs. 16.2%, *p* = 0.005), and chronic kidney disease (7.3 vs. 10.0%, *p* = 0.030). In addition, paroxysmal AF (52.9 vs. 44.5%, *p* < 0.001), atrial flutter at baseline (10.5 vs. 5.7%, *p* = 0.001), and AF-related symptoms (58.6 vs. 40.9%, *p* < 0.001) were more frequent in the RVR group. No significant difference was found in serum hemoglobin (13.9 ± 2.2 vs. 14.0 ± 2.0 g/dL, *p* = 0.515) or N-terminal prohormone of brain natriuretic peptide level (654.5 ± 2622.2 vs. 173.7 ± 885.1 pg/mL, *p* = 0.076). In baseline echocardiography, the RVR group revealed a lower left ventricular ejection fraction (55.9 ± 11.6 vs. 59.2 ± 10.1%, *p* < 0.001), which led to a higher proportion of heart failure (17.4 vs. 12.5%, *p* = 0.006) and heart failure with reduced ejection fraction (10.1 vs. 5.5%, *p* < 0.001). However, the RVR group exhibited a smaller left atrial diameter (44.4 ± 7.9 vs. 47.4 ± 10.7 mm, *p* < 0.001) and left atrial volume index (48.8 ± 22.7 vs. 56.5 ± 27.8 kg/m^2^, *p* < 0.001). Regarding the treatment strategy, the RVR group was more frequently treated with rate control agents (75.3 vs. 66.2%, *p* < 0.001) and received rhythm control (46.9 vs. 41.3%, *p* = 0.019), including antiarrhythmic drugs (41.0 vs. 31.3%, *p* < 0.001).

### 3.2. Clinical Outcomes

**Figure 2:**
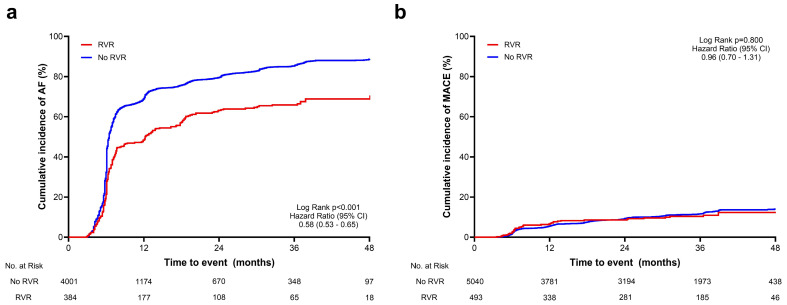
Time-to-event curves for primary and secondary endpoints. Figure above shows time-to-event curves for primary endpoint (recurrence of AF during follow-up) in the total cohort (**a**) and secondary endpoint (MACE) in the total cohort (**b**). RVR, rapid ventricular response; AF, atrial fibrillation; MACE, major adverse cardiovascular event; CI, confidence interval.

In 5533 patients, the mean follow-up duration was 28.6 ± 18.5 months—753 patients were lost to follow-up at 6 months. The primary endpoint was assessed in 4385 patients who had a follow-up 12-lead ECG (Table 2). Among them, 3600 (82.1%) had AF at least once during the cumulative ECG follow-up. Compared with patients without RVR, the RVR group exhibited significantly lower recurrence of AF during follow-up (hazard ratio: 0.58, 95% confidence interval [CI]: 0.53–0.65, *p* < 0.001; Figure 2a). Similarly, the RVR group had a lower incidence of secondary endpoints in manifestation of AF (i) at 6 months follow-up (50.7 vs. 73.4%, *p* < 0.001), (ii) at 12 months follow-up (45.6 vs. 72.4%, *p* < 0.001), and (iii) at 36 months follow-up (51.5 vs. 74.6%, *p* < 0.001; Table 2). 

All patients (n = 5533) were evaluated for occurrence of MACEs. There was no significant difference in the recurrence of MACEs (hazard ratio: 0.96, 95% CI: 0.70–1.31, *p* = 0.800; Figure 2b). Further analysis of propensity score-matched cohorts revealed consistent findings regarding the primary endpoint (hazard ratio: 0.61, 95% CI: 0.51–0.74, *p* < 0.001; Supplementary Figure S2).

The predictors of the primary endpoint were identified using multivariable Cox regression analysis (Table 3). The independent predictors included clinical factors that indicated AF progression: increased age and increase in left atrial diameter. Paroxysmal AF, atrial flutter at baseline ECG, AF-related symptoms, and rhythm control history were identified as protective factors against AF. AF with RVR was independently associated with the primary endpoint, resulting in a 39% decrease in the risk of AF recurrence during follow-up (adjusted hazard ratio: 0.61, 95% CI: 0.53–0.71, *p* < 0.001). 

### 3.3. Effect of Ventricular Rate on AF Recurrence

**Figure 3:**
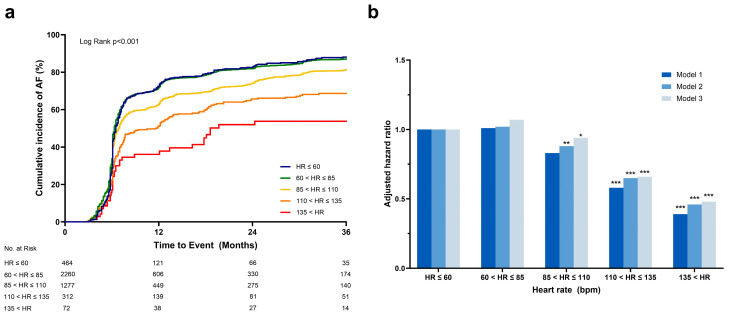
Association of primary endpoint and heart rate (subdivided into 5 groups). Figure above shows time-to-event curves for primary endpoint in the total cohort, divided by 5 groups according to baseline HR (**a**) and adjusted risk in three models (**b**). Model 1: Unadjusted. Model 2: Demographics (age, sex), comorbidities (history of valvular heart disease, heart failure, pacemaker implantation, history of stroke or TIA, chronic kidney disease), diastolic blood pressure, and AF-related factors (paroxysmal AF, atrial flutter at baseline, AF-related symptoms). Model 3: Demographics (age, sex), comorbidities (history of valvular heart disease, heart failure, pacemaker implant, history of stroke or TIA, chronic kidney disease), diastolic blood pressure, AF-related factors (paroxysmal AF, atrial flutter at baseline, AF-related symptoms), treatment (warfarin or coumarin, non-vitamin K antagonist oral anticoagulant, ACE inhibitor or angiotensin receptor blocker, rate control medication, rhythm control), and echocardiography (left atrial diameter, left ventricular ejection fraction)**.** (*) *p*-value < 0.05, (**) *p*-value < 0.01, (***) *p*-value < 0.001. AF, atrial fibrillation; HR, heart rate; TIA, transient ischemic attack.

**Figure 4:**
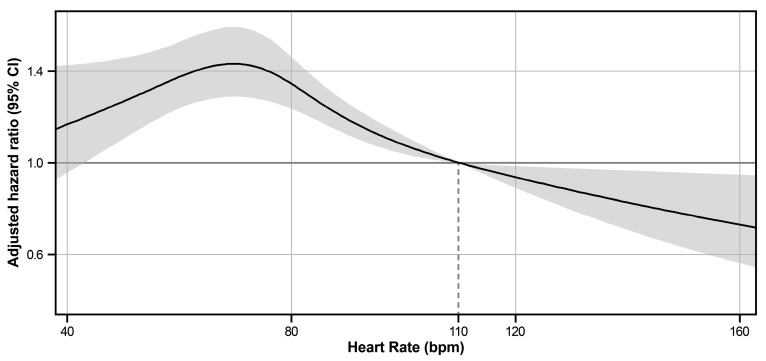
Adjusted hazard ratio according to heart rate. The adjusted hazard ratios and 95% confidence intervals according to heart rate were described using a cubic spline curve. Heart rate at 110 bpm was set as the reference for the adjusted hazard ratio. CI, confidence interval.

Patients were further subdivided into five groups according to their ventricular rate on baseline ECG: (i) heart rate ≤ 60, (ii) 60 < heart rate ≤ 85, (iii) 85 < heart rate ≤ 110, (iv) 110 < heart rate ≤ 135, and (v) 135 < heart rate. Baseline characteristics among the five groups revealed significant differences in demographic factors, comorbidities, AF-related factors, echocardiographic findings, and medications (Supplementary Table S2). The cumulative incidence of AF was highest in patients with a slow ventricular response (heart rate ≤ 60) and patients with a normal ventricular rate (60 < heart rate ≤ 85), followed by patients with a higher ventricular rate (85 < heart rate ≤ 110; Figure 3a). AF recurrence was markedly decreased in the RVR group and was lowest in patients with a heart rate > 135 (*p* < 0.001). Adjustment for covariates such as demographics and AF-related factors (Model 2) resulted in consistent findings (Supplementary Table S3, Figure 3b). Model 3 included further adjustments for the treatment strategies (medications and rhythm control) and echocardiographic data. In Model 3, the patients with the highest ventricular rate revealed the lowest risk of AF recurrence (adjusted hazard ratio: 0.48, 95% CI: 0.33–0.71, *p* < 0.001), followed by patients with the second highest ventricular rate (110 < heart rate ≤ 135; adjusted hazard ratio: 0.66, 95% CI: 0.54–0.80, *p* < 0.001). Consequently, ventricular rate was negatively associated with adjusted risk of AF recurrence, showing a reverse-J shaped curve (Figure 4).

### 3.4. Outcome in Special Populations

**Figure 5:**
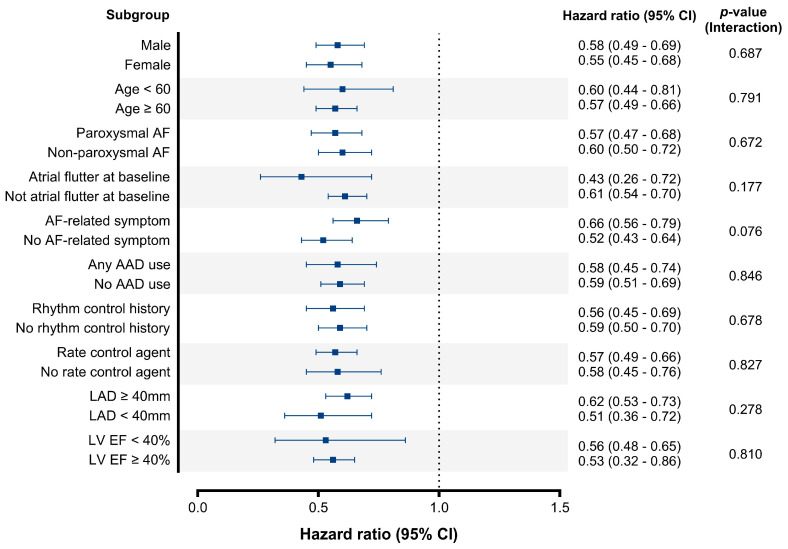
Subgroup analysis. Patients without rapid ventricular response were set as the reference in each group. CI, confidence interval; AF, atrial fibrillation; AAD, antiarrhythmic drug; LAD, left atrial diameter; LV EF, left ventricular ejection fraction.

In subgroup analysis, the effect of RVR on AF recurrence remained consistent, and no significant interaction was found in various subgroups (Figure 5).

To reduce selection bias, clinical outcomes were compared between patients who maintained sinus rhythm at baseline and those with AF (either RVR or no RVR). Patients with sinus rhythm at baseline revealed the lowest recurrence of AF and MACEs (Supplementary Figure S3). Furthermore, to minimize the impact of rhythm control and AF pattern on AF recurrence, sensitivity analysis was performed on patients without rhythm control (n = 3220) and those without persistent AF (n = 1499; Supplementary Table S4). The impact of AF with RVR on AF recurrence remained consistent in sensitivity analysis, which showed a lower recurrence of AF in the RVR group (Supplementary Table S5).

## 4. Discussion

Based on a multicenter, prospective cohort of patients with non-valvular AF, we investigated the impact of ventricular rate during AF on the clinical outcomes of rhythm control. Compared to patients without RVR at baseline, those with RVR had a lower recurrence of AF during follow-up, without a significant difference in MACEs. The risk of AF decreased as heart rate increased, which resulted in a negative relationship with heart rate. The association of RVR with AF recurrence was consistent in heart failure with reduced ejection fraction. Our findings were derived from a large-scale, validated cohort, and the outcomes from long-term follow-up were assessed.

### 4.1. Mechanisms

Several mechanisms may support the finding of lower AF recurrence in the case of a higher ventricular rate. First, long-standing persistent AF leads to functional remodeling of the atrioventricular (AV) node, with an increased frequency of tissue activation. Similarly, prolonged duration of AF is known to result in electrophysiological remodeling of the atrium and sinus nodes, leading to sinus node dysfunction [13]. Experimental studies have revealed that chronic high atrial rates also affect AV node function by increasing AV node conduction time and the AV node effective refractory period [14]. Thus, patients with chronic AF may exhibit poor AV nodal function and a slower ventricular rate.

Second, ventricular rate during AF may be associated with degree of cardiac remodeling. Cardiac fibrosis due to chronic oxidative stress and persistent low-grade inflammation (i.e., an aging heart) could also contribute to atrial fibrosis [15]. Cardiac fibrosis also influences the conduction system, including the AV node. Therefore, progressive cardiac fibrosis affects AV nodal function (i.e., the AV nodal effective refractory period) and atrial fibrosis, which may present as chronic AF with a slower ventricular rate. In contrast, AF with RVR may reflect less cardiac fibrosis progression, which supports better outcomes in this population. Conversely, heart rate could also affect the degree of atrial remodeling in slower heart rates. A heart rate below the normal range prolongs diastolic filling time, leading to higher filling pressures in the left atrium and ventricle [16]. Increased atrial afterload may promote atrial remodeling and dilatation, which contributes to the development and progression of AF [17]. In our cohort, patients with lower ventricular rates (heart rate ≤ 60) revealed significantly larger left atria, which reflects more progressed atrial remodeling that leads to higher recurrence of AF.

Lastly, increased activity of the cardiac autonomic nervous system may initiate AF with a high atrial fibrillatory rate and sustain rapid ventricular rate [8,18]. Proarrhythmic effects of the autonomic nervous system in AF with RVR indicate an earlier phase of atrial remodeling, with little role for structural substrates. In summary, AF with RVR may reflect the earlier phase of atrial myopathy, in which electrophysiological remodeling is ongoing without significant progression of structural remodeling. No difference having been observed in MACE occurrence may also be supported by the less progressed atrial remodeling in the RVR group as well as the younger age.

### 4.2. Clinical Implications

Ablation strategies for AF have shown remarkable advances over the past decades, leading to improved efficacy and fewer complications. However, the effect of catheter ablation is not consistent or optimal in the general AF population [19,20]. Therefore, rhythm control should be decided using an individualized approach, considering the clinical factors favorable for rhythm control, including younger age, paroxysmal AF, limited atrial modelling, and fewer comorbidities [2,21,22].

In this study, AF with RVR was identified as a favorable marker for the absence of AF recurrence. In the RVR group, more frequent AF-related symptoms and a higher proportion of heart failure might have led to a more active rhythm and rate control. Consequently, the lower recurrence of AF in patients with RVR may be owing to the mediating effect by confounders. However, the adjustment of confounding variables using different methods resulted in consistent outcomes. Thus, patients with AF and RVR may have better outcomes after active rhythm control. Nevertheless, the outcomes of rhythm control should not be solely determined by ventricular rate. For instance, patients with permanent AF may present with RVR that is intractable to medication and may necessitate AV node ablation. AF with RVR may also be common in patients with stressful conditions, such as critically ill conditions or advanced heart failure. Therefore, AF with RVR does not reflect a direct cause-and-effect relationship with a favorable response to rhythm control. Rather, AF with RVR is a favorable prognostic marker that should be comprehended with other relevant factors. In our study, the RVR group presented concomitant clinical factors that favor (or indicate) active rhythm control—younger age, paroxysmal AF, highly symptomatic AF, and smaller left atrium. In patients with RVR consisting of other favorable clinical factors, treatment strategies should not be limited to rate control; active and early rhythm control should also be considered.

### 4.3. Limitations

This study had several limitations. First, the true ventricular rate may have been underestimated because more than half of the patients were administered rate control medications at baseline. However, patients with RVR revealed a significantly higher proportion of rate control medications than patients without RVR (75.3 vs. 66.2%, *p* < 0.001), implying that these patients reflected (i) a higher ventricular rate or (ii) more persistent tachycardia that was not fully tolerated with current medication. This may have accentuated the distinct differences in outcomes between the two groups. Second, the CODE-AF registry was originally designed to evaluate the outcomes of medical treatment in patients with non-valvular AF, and the major outcomes were focused on non-rhythmic outcomes such as mortality, thromboembolic events, and major bleeding. For instance, factors related to atrial remodeling such as duration of AF were not specified. Also, continuous or ambulatory ECG monitoring was not included, which may underestimate the recurrence of AF. In addition, non-pharmacological rhythm control strategies performed after baseline, including electric cardioversion and catheter ablation, were not specified. Patients with RVR may have received more aggressive rhythm control during follow-up, which was not adjusted for. Nonetheless, adjustment for possible confounding variables and various sensitivity analyses resulted in a significantly lower risk of AF in the RVR group, which was consistent across various time periods (i.e., 6, 12, and 36 months of follow-up). Third, the primary endpoint was defined as a binary outcome of AF recurrence (recurrence or no recurrence) based on 12-lead ECG, which provides limited evidence to estimate the AF burden. Also, the binary outcome judged by a single ECG may be highly influenced by the AF pattern (paroxysmal vs. persistent AF). To overcome this bias, additional analysis excluding persistent AF was carried out, which revealed the consistent association of RVR and AF recurrence. Yet the changing trend in the outcome—from binary AF detection by a single ECG to quantification of AF burden—should be perceived and pursued as the therapeutic target [23]. Further research is also needed to clarify the effect of AF burden on clinical outcomes. Finally, this study was limited to the East Asian population, which exclusively comprised South Korean patients with non-valvular AF. Further evidence is needed to generalize our findings to patients with AF of various ethnicities and conditions.

## 5. Conclusions

Patients with AF and RVR had a significantly lower recurrence of AF without an increase in MACEs. An increase in ventricular rate revealed a negative association with the risk of AF recurrence. Patients with AF and RVR should be considered for early rhythm control in addition to rate control, and an integrated approach should be applied in the decision to pursue rhythm control.

## Figures and Tables

**Table 1:** Baseline characteristics.

	Total (n = 5533)	RVR (n = 493)	No RVR (n = 5040)	*p*-Value
Age (years)	68.1 ± 10.6	66.9 ± 11.4	68.1 ± 10.4	0.018
Sex (male)	3689 (66.7)	292 (59.2)	3397 (67.4)	<0.001
Systolic blood pressure (mmHg)	122.3 ± 16.0	121.6 ± 16.5	122.4 ± 16.0	0.321
Diastolic blood pressure (mmHg)	76.3 ± 12.7	78.0 ± 13.7	76.1 ± 12.6	0.002
Body mass index (kg/m^2^)	24.9 ± 3.5	24.9 ± 3.3	24.9 ± 3.5	0.791
CHA_2_DS_2_-VASc score	2.7 ± 1.7	2.7 ± 1.6	2.7 ± 1.6	0.447
HAS-BLED score	1.8 ± 1.0	1.6 ± 1.0	1.8 ± 1.0	<0.001
Hypertension	3691 (66.8)	310 (62.9)	3381 (67.1)	0.064
Diabetes mellitus	1484 (26.8)	128 (26.0)	1356 (26.9)	0.649
History of myocardial infarction	119 (2.2)	9 (1.8)	110 (2.2)	0.602
History of valvular heart disease	666 (12.0)	49 (9.9)	67 (12.2)	0.107
Surgery for valvular heart disease	20 (0.4)	1 (0.2)	19 (0.4)	0.539
Heart failure	718 (13.0)	86 (17.4)	632 (12.5)	0.006
Heart failure with reduced ejection fraction	264 (5.9)	39 (10.1)	225 (5.5)	<0.001
ICD implantation	51 (0.9)	1 (0.2)	50 (1.0)	0.001
Pacemaker implantation	180 (3.3)	12 (2.4)	168 (3.3)	0.224
Peripheral artery disease	273 (4.9)	21 (4.3)	252 (5.0)	0.469
History of stroke or transient ischemic attack	872 (15.8)	58 (11.8)	814 (16.2)	0.005
Dyslipidemia	1669 (30.2)	134 (27.2)	1535 (30.5)	0.121
Chronic kidney disease	541 (9.8)	36 (7.3)	505 (10.0)	0.030
Cancer	536 (9.7)	38 (7.7)	498 (9.9)	0.089
Current smoker	536 (9.7)	57 (11.6)	479 (9.5)	0.171
Current drinker	1203 (21.7)	115 (23.3)	1088 (21.5)	0.516
Paroxysmal AF	2402 (45.2)	261 (52.9)	2241 (44.5)	<0.001
Atrial flutter at baseline ECG	341 (6.2)	52 (10.5)	289 (5.7)	0.001
AF-related symptoms	2350 (42.5)	289 (58.6)	2061 (40.9)	<0.001
Hemoglobin (g/dL)	14.0 ± 2.0	13.9 ± 2.2	14.0 ± 2.0	0.515
NT-proBNP (pg/mL)	228.6 ± 1222.6	654.5 ± 2622.2	173.7 ± 885.1	0.076
Echocardiography				
Left atrial diameter (mm)	47.1 ± 10.5	44.4 ± 7.9	47.4 ± 10.7	<0.001
Left atrial volume index (kg/m^2^)	55.9 ± 27.5	48.8 ± 22.7	56.5 ± 27.8	<0.001
Left ventricular ejection fraction (%)	58.8 ± 10.2	55.9 ± 11.6	59.2 ± 10.1	<0.001
Treatment				
Warfarin or Coumadin	966 (17.5)	59 (12.0)	907 (18.0)	<0.001
Non-vitamin K antagonist oral anticoagulant	3673 (66.4)	348 (70.6)	3325 (66.0)	0.033
ACE inhibitor or angiotensin receptor blocker	2204 (39.8)	175 (35.5)	2029 (40.3)	0.036
Rate control agent	3709 (67.0)	371 (75.3)	3338 (66.2)	<0.001
Beta-blocker	3048 (55.1)	295 (59.8)	2753 (54.6)	0.025
Calcium channel blocker	1454 (26.3)	113 (22.9)	1341 (26.6)	0.065
Non-dihydropyridine calcium channel blocker	552 (10.0)	61 (12.4)	491 (9.7)	0.089
Digoxin	639 (11.5)	92 (18.7)	547 (10.9)	0.072
Rhythm control history	2313 (41.8)	231 (46.9)	2082 (41.3)	0.019
Previous catheter ablation	411 (7.4)	43 (8.7)	368 (7.3)	0.274
Previous direct current cardioversion	759 (13.7)	41 (8.3)	718 (14.2)	<0.001
Any antiarrhythmic drug	1778 (32.1)	202 (41.0)	1576 (31.3)	<0.001
Amiodarone	637 (11.5)	49 (9.9)	588 (11.7)	0.225

RVR, rapid ventricular response; ICD, implantable cardioverter defibrillator; AF, atrial fibrillation; ECG, electrocardiography; NT-proBNP, N-terminal prohormone of brain natriuretic peptide.

**Table 2:** Primary endpoint and secondary endpoints.

	Total (n = 5533)	RVR (n = 493)	No RVR (n = 5040)	*p*-Value
Primary endpoint: recurrence of AF				
N	4385	384	4001	
	3600 (82.1)	242 (63.0)	3358 (83.9)	<0.001
Secondary endpoints				
Manifestation of AF				
6 months				
N	3873	345	3528	
AF present	2763 (71.3)	175 (50.7)	2588 (73.4)	<0.001
12 months				
N	3387	283	3104	
AF present	2376 (70.2)	129 (45.6)	2247 (72.4)	<0.001
36 months				
N	2124	167	1957	
AF present	1545 (72.8)	86 (51.5)	1459 (74.6)	<0.001
AF-related hospitalization	1842 (33.3)	169 (34.3)	1673 (33.2)	0.661
MACE	523 (9.5)	42 (8.5)	481 (9.5)	0.508
Ischemic stroke	107 (1.9)	8 (1.6)	105 (2.1)	0.512
Systemic thromboembolism	7 (0.1)	1 (0.2)	6 (0.1)	0.617
Transient ischemic attack	11 (0.2)	1 (0.2)	10 (0.2)	0.983
Major bleeding	413 (7.5)	37 (7.5)	444 (8.8)	0.399
Myocardial infarction	18 (0.3)	2 (0.4)	17 (0.3)	0.814
Death	6 (0.1)	0 (0.0)	6 (0.1)	0.443

Chi-square test or Fisher’s exact test were used to compare RVR versus No RVR groups. RVR, rapid ventricular response; AF, atrial fibrillation; MACE, major adverse cardiovascular event.

**Table 3:** Predictors for AF recurrence.

Variables	Adjusted Hazard Ratio	95% Confidence Interval	*p*-Value
AF with RVR	0.613	0.528–0.713	<0.001
Sex (male)	1.018	0.940–1.103	0.656
Age (years)	1.014	1.010–1.018	<0.001
Diastolic blood pressure (mmHg)	1.000	0.997–1.003	0.899
Heart failure	0.977	0.865–1.104	0.707
ICD implant	1.460	1.050–2.032	0.025
History of stroke or transient ischemic attack	1.074	0.978–1.180	0.135
Chronic kidney disease	1.149	1.025–1.288	0.017
Paroxysmal AF	0.891	0.827–0.959	0.002
Atrial flutter at baseline ECG	0.792	0.697–0.902	<0.001
AF-related symptoms	0.808	0.750–0.871	<0.001
Left atrial diameter (mm)	1.003	1.002–1.005	<0.001
Left ventricular ejection fraction (%)	0.997	0.993–1.001	0.200
Warfarin or Coumadin use	1.143	1.042–1.254	0.005
Rate control agent	1.090	1.007–1.180	0.034
Rhythm control history	0.680	0.625–0.738	<0.001

AF, atrial fibrillation; RVR, rapid ventricular response; ICD, implantable cardioverter defibrillator; ECG, electrocardiography.

## Data Availability

The data underlying this article are available in the article and in its Supplementary Materials.

## Supplementary Materials

The following supporting information can be downloaded at: https://www.mdpi.com/article/10.3390/jcm13185469/s1, Figure S1: Plot for absolute standardized mean differences. Figure S2: Time-to-event curves after propensity score matching. Figure S3: Time-to-event curves including patients with sinus rhythm at baseline. Table S1: Definition of variables. Table S2: Baseline characteristics according to heart rate. Table S3: Impact of heart rate on AF recurrence. Table S4. Study population for sensitivity analysis. Table S5: Impact of AF with RVR on AF recurrence (sensitivity analysis).

## Abbreviations

AF	Atrial fibrillation
AV	Atrioventricular
CI	Confidence interval
ECG	Electrocardiography
MACE	Major adverse cardiovascular event
RVR	Rapid ventricular response
