# Supplementary material for: Impact of Atrial Fibrillation with Rapid Ventricular Response on Atrial Fibrillation Recurrence: From the CODE-AF Registry
Source: J Clin Med. 2024 Sep 14;13(18):5469. doi: 10.3390/jcm13185469 (PMC11432096; doi:10.3390/jcm13185469)
Supplement: Supplementary file 1 [file jcm-13-05469-s001.zip › jcm-3196864-supplementary.pdf]

Supplemental Materials

Supplementary Table S1. Definition of variables.

Variables	Definition
Dyslipidaemia	Total cholesterol > 200 mg/dL or previous use of statins
Chronic kidney disease	Estimated glomerular filtration rate < 60
Atrial flutter at baseline ECG	Atrial flutter at baseline ECG or Holter ECG (if atrial flutter was dominant during Holter ECG)
AF-related symptom	Any of six symptoms (palpitations, fatigue, dizziness, chest pain, and anxiety) during AF from the European Heart Rhythm Association scale 2a to 4
Rate control medications	Use of either beta-blockers, non-dihydropyridine calcium channel blockers, or digoxin
Rhythm control history	Any attempt at rhythm control that had been conducted or was conducted at the baseline, including catheter ablation (radiofrequency catheter ablation or cryoballoon ablation), direct current cardioversion, and antiarrhythmic drugs

AF, atrial fibrillation; ECG, electrocardiography.

Supplementary Table S2. Baseline characteristics according to heart rate.

	HR ≤ 60 (n=576)	60 < HR ≤ 85 (n=2,829)	85 < HR ≤ 110 (n=1,635)	110 < HR ≤ 135 (n=395)	135 < HR (n=98)	p-Value
Age (years)	68.7 ± 10.5	68.4 ± 10.3	67.6 ± 10.7	66.9 ± 11.1	66.9 ± 12.8	0.009
Sex (male)	412 (71.5)	1,925 (68.0)	1,060 (64.8)	241 (61.0)	51 (52.0)	<0.001
Systolic blood pressure (mmHg)	123.4 ± 16.9	122.1 ± 15.4	122.4 ± 16.6	121.8 ± 16.4	120.9 ± 17.3	0.389
Diastolic blood pressure (mmHg)	72.5 ± 12.0	75.7 ± 12.1	78.2 ± 13.2	78.2 ± 13.4	77.2 ± 14.7	<0.001
Body mass index (kg/m^2)	24.7 ± 3.6	24.9 ± 3.4	25.0 ± 3.6	24.9 ± 3.3	24.7 ± 3.5	0.284
CHA_2DS_2-VASc score	2.8 ± 1.7	2.7 ± 1.7	2.7 ± 1.7	2.6 ± 1.6	2.9 ± 1.7	0.490
HAS-BLED score	2.0 ± 1.1	1.8 ± 1.1	1.8 ± 1.0	1.6 ± 1.1	1.7 ± 0.9	<0.001
Hypertension	400 (69.4)	1,880 (66.5)	1,101 (67.3)	247 (62.5)	63 (64.3)	0.222
Diabetes mellitus	146 (25.3)	741 (26.2)	469 (28.7)	102 (25.8)	26 (26.5)	0.362
History of myocardial infarction	12 (2.1)	63 (2.2)	35 (2.1)	8 (2.0)	1 (1.0)	0.949
History of valvular heart disease	98 (17.0)	344 (12.2)	175 (10.7)	43 (10.9)	6 (6.1)	0.001
Surgery for valvular heart disease	2 (0.3)	13 (0.5)	4 (0.2)	1 (0.3)	0 (0.0)	0.761
Heart failure	64 (11.1)	323 (11.4)	245 (15.0)	65 (16.5)	21 (21.4)	<0.001
ICD implantation	8 (1.4)	30 (1.1)	12 (0.7)	1 (0.3)	0 (0.0)	0.244
Pacemaker implantation	39 (6.8)	92 (3.3)	37 (2.3)	11 (2.8)	1 (1.0)	<0.001
Peripheral artery disease	36 (6.2)	148 (5.2)	68 (4.2)	19 (4.8)	2 (2.0)	0.164
History of stroke or transient ischemic attack	110 (19.1)	475 (16.8)	229 (14.0)	44 (11.1)	14 (14.3)	0.002
Dyslipidemia	200 (34.7)	860 (30.4)	475 (29.1)	106 (26.8)	28 (28.6)	0.063
Chronic kidney disease	80 (13.9)	261 (9.2)	164 (10.0)	28 (7.1)	8 (8.2)	0.004
Cancer	56 (9.7)	280 (9.9)	162 (9.9)	31 (7.8)	7 (7.1)	0.647
Current smoker	53 (9.2)	264 (9.3)	162 (9.9)	44 (11.1)	13 (13.3)	0.551
Current drinker	170 (29.5)	861 (30.4)	496 (30.3)	125 (31.6)	28 (28.6)	0.758
Paroxysmal AF	278 (48.3)	1,189 (42.0)	774 (47.3)	199 (50.4)	62 (63.3)	<0.001
Atrial flutter at baseline ECG	47 (8.2)	146 (5.2)	96 (5.9)	43 (10.9)	9 (9.2)	<0.001
AF-related symptoms	224 (38.9)	1,108 (39.2)	729 (44.6)	223 (56.5)	66 (67.3)	<0.001

Echocardiography						
Left atrial diameter (mm)	49.1 ± 8.6	47.7 ± 12.3	46.4 ± 7.9	44.6 ± 8.2	43.8 ± 6.5	<0.001
Left atrial volume index (kg/m^2)	60.7 ± 29.6	57.3 ± 29.2	53.5 ± 23.8	48.9 ± 22.8	48.5 ± 22.6	<0.001
Left ventricular ejection fraction (%)	61.3 ± 10.0	59.7 ± 9.5	57.5 ± 10.8	56.2 ± 11.4	54.8 ± 12.7	<0.001
Treatment						
Warfarin or Coumadin	114 (19.8)	540 (19.1)	253 (15.5)	52 (13.2)	7 (7.1)	<0.001
Non-vitamin K antagonist oral anticoagulant	363 (63.0)	1,852 (65.5)	1,110 (67.9)	277 (70.1)	71 (72.4)	0.045
ACE inhibitor or angiotensin receptor blocker	264 (45.8)	1,133 (40.0)	632 (38.7)	139 (35.2)	36 (36.7)	0.009
Rate control agent	287 (49.8)	1,880 (66.5)	1,171 (71.6)	297 (75.2)	74 (75.5)	<0.001
Beta-blocker	229 (39.8)	1,539 (54.4)	985 (60.2)	241 (61.0)	54 (55.1)	<0.001
Calcium channel blocker	158 (27.4)	804 (28.4)	379 (23.2)	87 (22.0)	26 (26.5)	0.001
Non-dihydropyridine calcium channel blocker	48 (8.3)	292 (10.3)	151 (9.2)	45 (11.4)	16 (16.3)	0.078
Digoxin	50 (8.7)	295 (10.4)	202 (12.4)	67 (17.0)	25 (25.5)	<0.001
Rhythm control history	203 (35.2)	1,191 (42.1)	688 (42.1)	191 (48.4)	40 (40.8)	0.002
Previous catheter ablation	43 (7.5)	218 (7.7)	107 (6.5)	37 (9.4)	6 (6.1)	0.333
Previous direct current cardioversion	66 (11.5)	459 (16.2)	193 (11.8)	37 (9.4)	4 (4.1)	<0.001
Any antiarrhythmic drug	145 (25.2)	867 (30.6)	564 (34.5)	163 (41.3)	39 (39.8)	<0.001
Amiodarone	62 (10.8)	351 (12.4)	175 (10.7)	40 (10.1)	9 (9.2)	0.302

HR, heart rate; ICD, implantable cardioverter defibrillator; AF, atrial fibrillation; ECG, electrocardiography.

Supplementary Table S3. Impact of heart rate on AF recurrence.

	HR ≤ 60 (n=576)	60 < HR ≤ 85 (n=2,829)	p-Value	85 < HR ≤ 110 (n=1,635)	p-Value	110 < HR ≤ 135 (n=395)	p-Value	135 < HR (n=98)	p-Value
**Model 1**	1 (Reference)	1.009 (0.907–1.124)	0.865	0.832 (0.741–0.933)	0.002	0.584 (0.493–0.690)	<0.001	0.395 (0.280–0.558)	<0.001
**Model 2**	1 (Reference)	1.019 (0.913–1.138)	0.738	0.882 (0.783–0.993)	0.038	0.649 (0.545–0.772)	<0.001	0.465 (0.328–0.658)	<0.001
**Model 3**	1 (Reference)	1.071 (0.949–1.209)	0.266	0.940 (0.823–1.073)	0.357	0.660 (0.543–0.802)	<0.001	0.484 (0.330–0.709)	<0.001

Risk of AF recurrence was described as adjusted hazard ratio (95% confidence interval).

Model 1: Unadjusted.

Model 2: Demographics (age, sex), comorbidities (history of valvular heart disease, heart failure, pacemaker implantation, history of stroke or TIA, chronic kidney disease), diastolic blood pressure, and AF-related factors (paroxysmal AF, atrial flutter at baseline, AF-related symptoms).

Model 3: demographics (age, sex), comorbidities (history of valvular heart disease, heart failure, pacemaker implant, history of stroke or TIA, chronic kidney disease), diastolic blood pressure, AF-related factors (paroxysmal AF, atrial flutter at baseline, AF-related symptoms), treatment (warfarin or coumarin, non-vitamin K antagonist oral anticoagulant, ACE inhibitor or angiotensin receptor blocker, rate control medication, rhythm control), and echocardiography (left atrial diameter, left ventricular ejection fraction)

AF, atrial fibrillation; HR, heart rate; TIA, transient ischemic attack.

Supplementary Table S4. Study population for sensitivity analysis.

Cohort	Included Patients	Excluded Patients
Total cohort	n=5,533	
Cohort A: Exclusion of patients treated with rhythm control	n=3,220	n=2,313
Cohort B: Exclusion of patients with persistent AF (from Cohort A)	n=1,499	n=1,721

AF, atrial fibrillation.

Supplementary Table S5. Impact of AF with RVR on AF recurrence (sensitivity analysis).

	Total Cohort (n=5,533)		Cohort A (n=3,220)		Cohort B (n=1,499)	
	**Model 1**	**Model 3**	**Model 1**	**Model 3**	**Model 1**	**Model 3**
**No RVR**	1 (Reference)	1 (Reference)	1 (Reference)	1 (Reference)	1 (Reference)	1 (Reference)
**RVR**	0.577 (0.507-0.658)	0.622 (0.536-0.721)	0.583 (0.494-0.687)	0.636 (0.527-0.769)	0.582 (0.458-0.740)	0.589 (0.436-0.796)
**p-value**	<0.001	<0.001	<0.001	<0.001	<0.001	<0.001

Risk of AF recurrence was described as adjusted hazard ratio (95% confidence interval).

Model 1: Unadjusted.

Model 3: demographics (age, sex), comorbidities (history of valvular heart disease, heart failure, pacemaker implant, history of stroke or TIA, chronic kidney disease), diastolic blood pressure, AF-related factors (paroxysmal AF, atrial flutter at baseline, AF-related symptoms), treatment (warfarin or coumarin, non-vitamin K antagonist oral anticoagulant, ACE inhibitor or angiotensin receptor blocker, rate control medication, rhythm control), and echocardiography (left atrial diameter, left ventricular ejection fraction).

AF, atrial fibrillation; RVR, rapid ventricular response.

Supplementary Figure S1. Plot for absolute standardized mean differences.

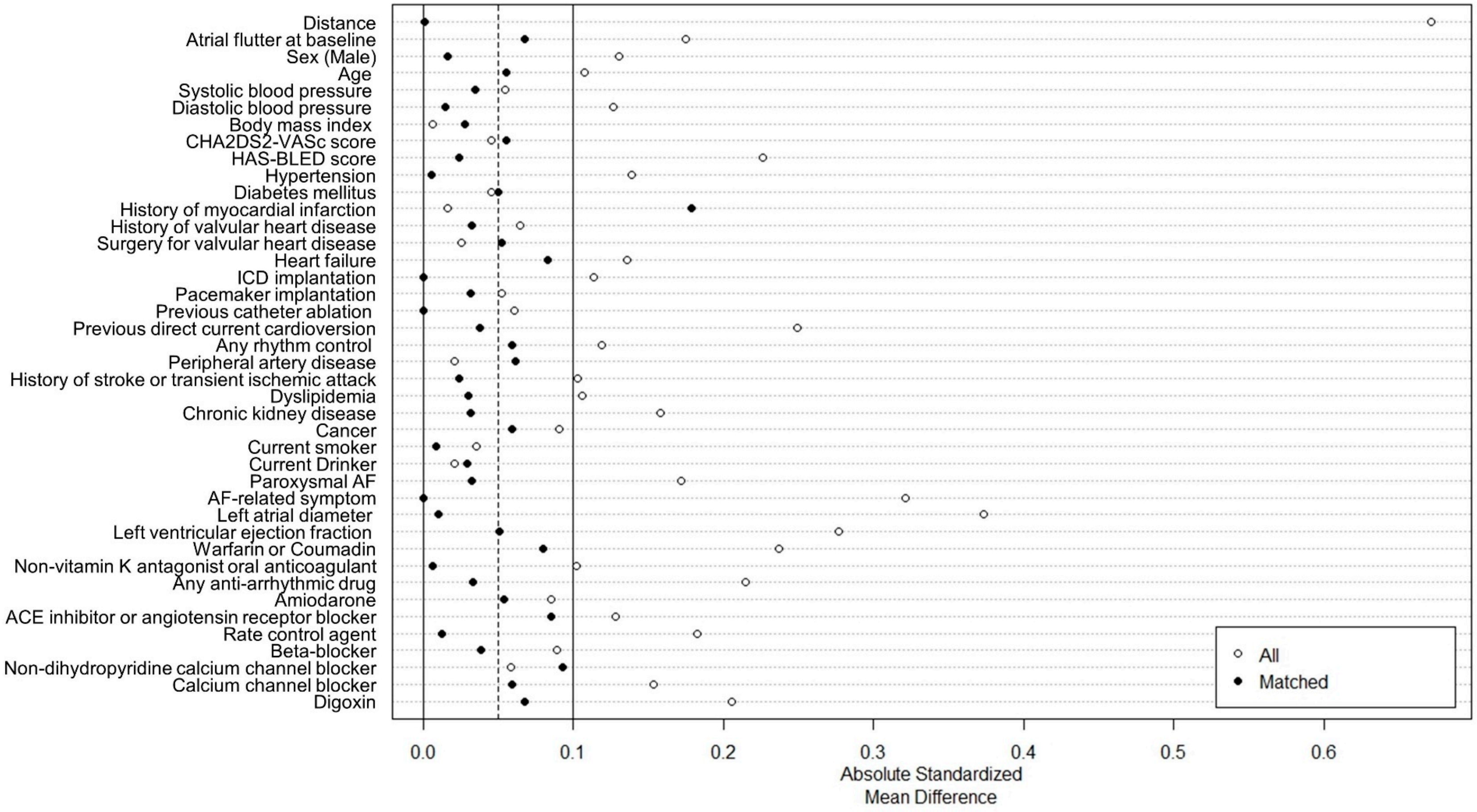

AF, atrial fibrillation; ICD, implantable cardioverter defibrillator.

Supplementary Figure S2. Time-to-event curves after propensity score matching.

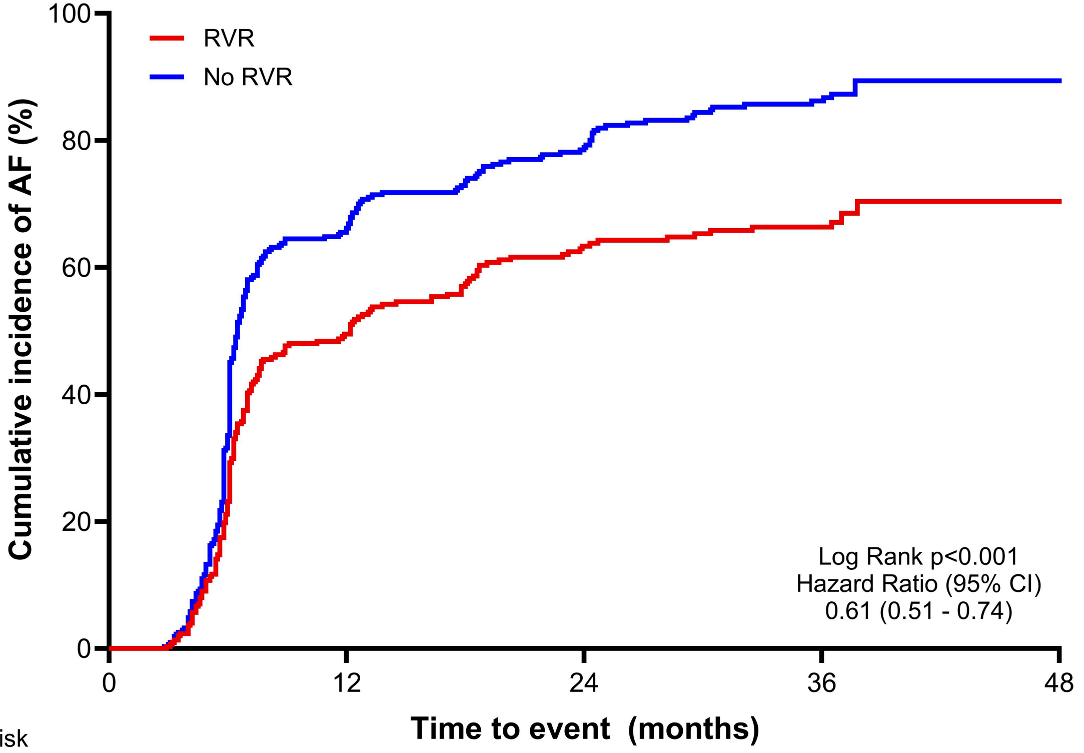

No. at Risk					
No RVR	310	101	56	26	4
RVR	300	135	83	50	12

Figure above shows time-to-event curves for primary endpoint after propensity score matching.

RVR, rapid ventricular response; AF, atrial fibrillation; CI, confidence interval.

Supplementary Figure S3. Time-to-event curves, including patients with sinus rhythm at baseline.

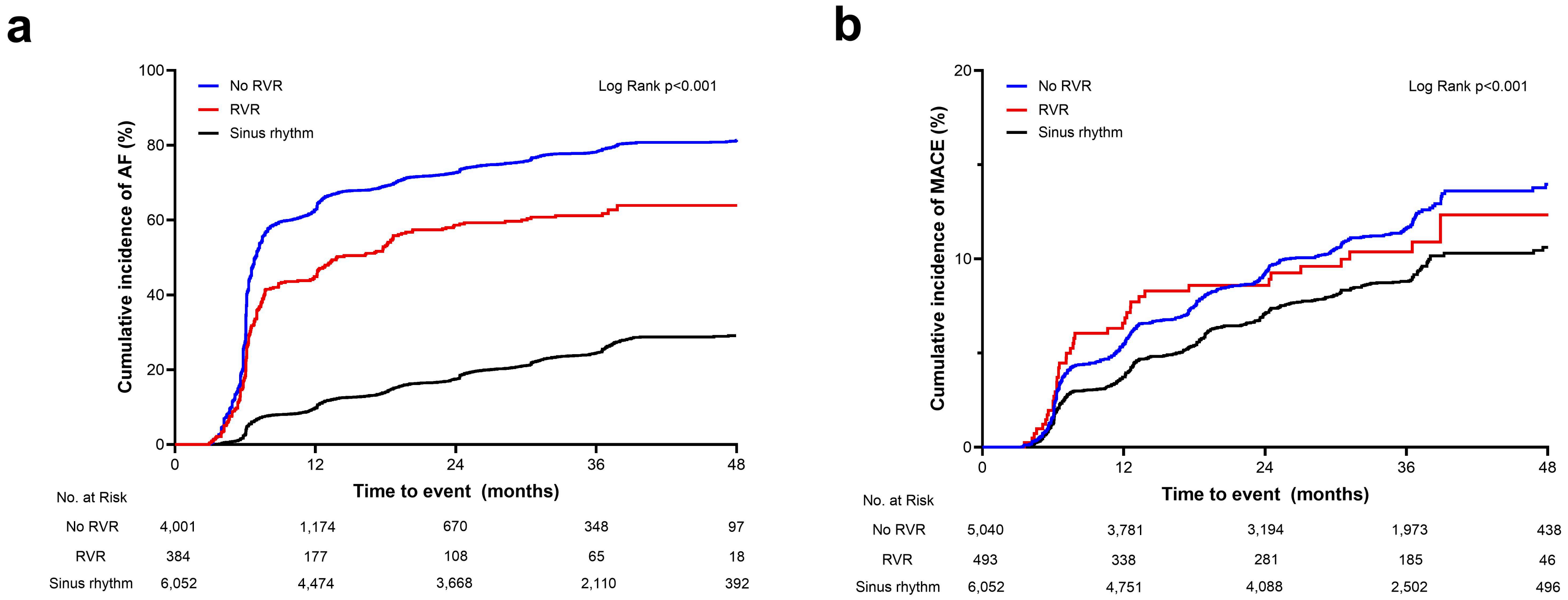

RVR, rapid ventricular response; AF, atrial fibrillation; MACE, major adverse cardiovascular event; CI, confidence interval.
